# United States Veterinary Medical Association

**Published:** 1894-02

**Authors:** Rush S. Huidekoper, Nelson P. Hinkley

**Affiliations:** President; Secretary


### UNITED STATES VETERINARY MEDICAL ASSOCIATION.

### OFFICERS 1893–94.

| | |
|---|---|
| W. Horace Hoskins, President | 34-5254 Ludlow Street, Philadelphia, Pa. |
| A. W. Clement, Vice-President | 902 Cathedral Street, Baltimore, Md. |
| T. J. Turner, Secretary | Columbia, Missouri |
| Jas. L. Robertson, Treasurer | 409 9th Ave., N. Y. City |

### COMMITTEES.

### *Comitia Minora.*

| | |
|---|---|
| (Ex-officio) W. Horace Hoskins, President | 3452-54 Ludlow Street, Philadelphia, Pa. |
| (Ex-officio) A. W. Clement, Vice President | 902 Cathedral Street, Baltimore, Md. |
| (Ex-officio) T. J. Turner Secretary | Columbia, Missouri |
| (Ex-officio) Jas. L. Robertson, Treasurer | 409 9th Ave , N. Y. City |
| A. Liautard, Chairman | 141 W. 54th Street, N. Y. City |
| W. L. Williams | Bozeman, Montana |
| R. S. Huidekoper | 155 West 56th Street. N. Y. City |
| T. B. Rayner | Chestnut Hill, Philadelphia, Pa. |
| F. H Osgood | 50 Village Street, Boston, Massachusetts |
| J. F. Winchester | Lawrence, Massachusetts |
| A. H. Baker | 145 Michigan Ave., Chicago, Ill. |

### *Intelligence and Education.*

| | |
|---|---|
| C. A. Cary, Chairman | Auburn, Alabama |
| Nelson P. Hinkley | 395 Ellicott Street, Buffalo, N. Y. |
| E. A. A. Grange | Agricultural College, Michigan |
| J. C. Meyer, Jr. | 379-81 Walnut Street, Cincinnati, Ohio |

### *Finance.*

| | |
|---|---|
| Wm. Dougherty, Chairman | 1035 Cathedral Street, Baltimore, Md. |
| S. S. Baker | 901 Jackson Boulevard, Chicago, Illinois |
| R. A. McLean | 14 and 16 Nevins St., Brooklyn, N. Y. |

### *Committee on Diseases.*

| | |
|---|---|
| Leonard Pearson, Chairman | 2200 Pine St., Philadelphia, Pa. |
| N. S. Mayo | Manhattan, Kansas |
| S. E. Weber | Lancaster, Pa. |
| Wyatt Johnston | 244 Mountain Street, Montreal, Canada |
| T. D. Hinebauch | Fargo, North Dakota |

### *Prize.*

| | |
|---|---|
| R. S. Huidekoper, Chairman | 155 West 56th St., N. Y. City |
| W. H. Lowe | 190 Ellison Street, Paterson, N. J. |
| L. McLean | 14 and 16 Nevins Street, Brooklyn, N. Y. |

### *Army Legislation.*

| | |
|---|---|
| F. L. Kilborne, Chairman | Department of Agriculture, Washington, D. C. |
| Gerald E. Griffin | Fort McIntosh, Laredo, Texas |
| James A. Waugh | 49 Race Street, Allegheny, Pa |

*Publication Committee.*

| | |
|---|---|
| L. H. Howard, Chairman | 1440 Washington St., Boston, Mass. |
| J. B. Rayner | West Chester, Pa. |
| A. T. Sellers | 312 South 5th Street, Camden, N. J. |

SPECIAL COMMITTEES.

*Congress of Colleges.*

| | |
|---|---|
| W. Horace Hoskins, Chairman | 3452-54 Ludlow Street, Philadelphia, Pa. |
| A. W. Clement | 902 Cathedral Street, Baltimore, Md. |
| John W. Gadsden | 128 North 10th Street, Philadelphia, Pa. |

*Act of Incorporation.*

| | |
|---|---|
| C. P Lyman, Chairman | 50 Village Street, Boston, Massachusetts |
| Wm. Dougherty | 1035 Cathedral Street, Baltimore, Md. |
| D. J. Dixon | 366 Washington Street, Hoboken, N. J. |

*Revision of Constitution and By-Laws.*

| | |
|---|---|
| W. Horace Hoskins, President | 3452-54 Ludlow Street, Philadelphia, Pa. |
| A W. Clement, Vice-President | 902 Cathedral Street, Baltimore, Md. |
| T. J. Turner, Secretary | Columbia, Missouri |
| Jas. L. Robertson, Treasurer | 409 9th Avenue, N. Y. City |
| W. B. E Miller, Chairman | 527 Penn Street, Camden, N J. |
| Leonard Pearson | 2200 Pine Street, Philadelphia, Pa. |
| S. Stewart | 7½ St James St., Kansas City, Kansas |

*Nomenclature of Swine Plague and Hog Cholera.*

| | |
|---|---|
| A. W. Clement, Chairman | 902 Cathedral Street Baltimore, Md. |
| Austin Peters | 35 Congress Street, Boston, Mass. |
| Theobald Smith | 1804 Columbia Road, Washington, D. C. |

