# Correction

**Published:** 1894-03

**Authors:** T. J. Turner

**Affiliations:** Secretary U. S. V. M. A.


### CORRECTION.

In looking over the published list of officers and committees in the February number of your journal, I note that one mistake occurs, namely, that of Dr. E. A. A. Grange on the Committee of Intelligence and Education, U. S. V. M. A. This place is filled by Dr. W. B. Niles, of Ames, Iowa.

Yours truly,

T. J. TURNER, Secretary U. S. V. M. A.

---

### N. H. PAAREN, M.D.

The many veterinarians who have been connected with the Bureau of Animal Industry and other friends of Dr. N. H. Paaren will regret to hear that ill-health has obliged him to leave for warmer climates, Los Angeles, Cal., Mexico and the West Indies, where he expects to remain indefinitely.

